# Supplementary material for: Timing and cell specificity of senescence drives postnatal lung development and injury
Source: Nat Commun. 2023 Jan 17;14:273. doi: 10.1038/s41467-023-35985-4 (PMC9845377; doi:10.1038/s41467-023-35985-4)
Supplement: Supplementary file 1 — Supplementary Information [file 41467_2023_35985_MOESM1_ESM.pdf]

Supplemental Information

Timing and cell specificity of senescence drives postnatal lung development and injury

Hongwei Yao[1*], Joselynn Wallace[2], Abigail L. Peterson[1], Alejandro Scaffa[1], Salu Rizal[1], Katy Hegarty[1], Hajime Maeda[1], Jason L. Chang[1], Nathalie Oulhen[1], Jill A. Kreiling[1], Kelsey E. Huntington[3], Monique E. De Paepe[4], Guilherme Barbosa[1], Phyllis A. Dennery[1,5*]

[1]Department of Molecular Biology, Cell Biology & Biochemistry, Division of Biology and Medicine, Brown University, Providence, RI 02912, USA;

[2]Center for Computational Biology of Human Disease and Center for Computation and Visualization, Brown University, Providence, RI 02912, USA;

[3]Department of Pathology and Laboratory Medicine, Warren Alpert Medical School, Brown University, Providence, RI 02903, USA;

[4]Department of Pathology, Women and Infants Hospital, Providence, RI 02905, USA;

[5]Department of Pediatrics, Warren Alpert Medical School of Brown University, Providence, RI 02903, USA

*** These authors jointly supervised this work:**

Hongwei Yao, email: hongwei_yao@brown.edu

Phyllis Dennery, email: phyllis_dennery@brown.edu

Running title: Senescence in postnatal lung development and injury

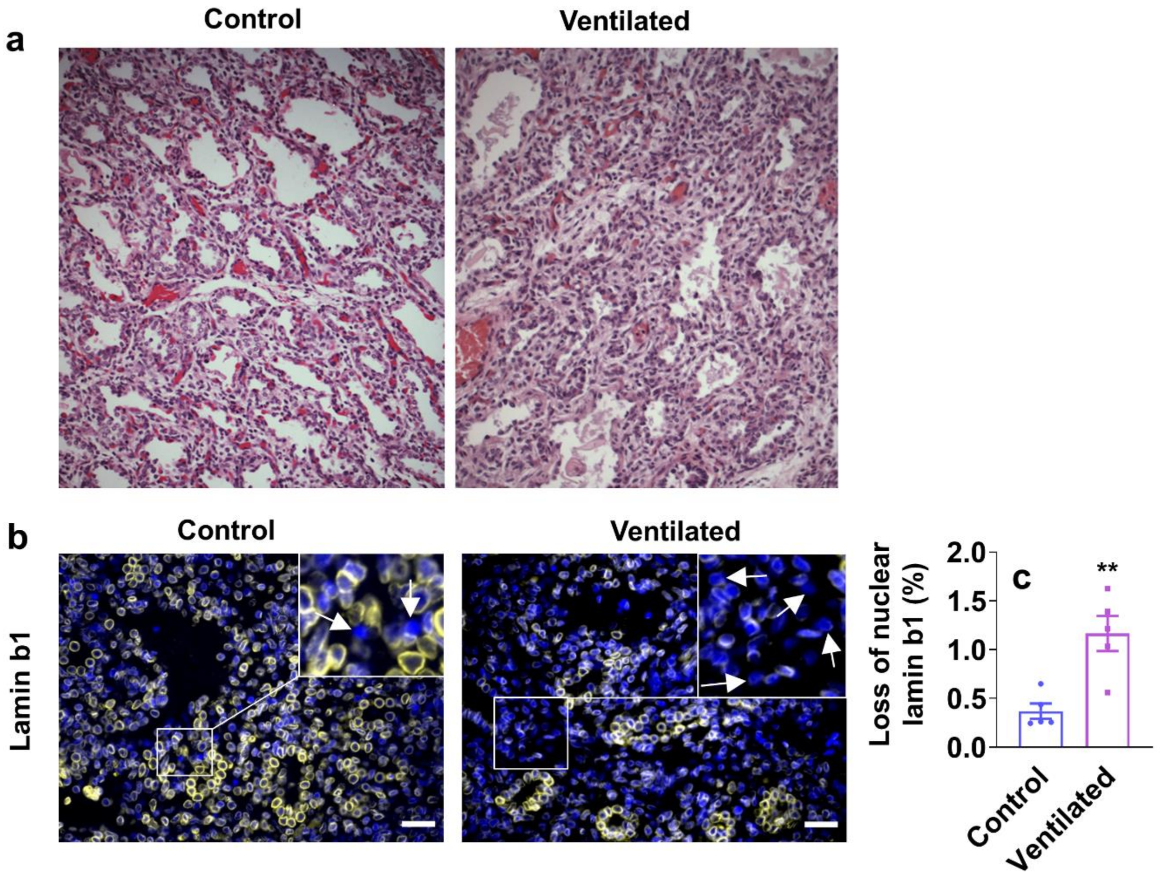

Supplementary Figure 1. Nuclear lamin b1 loss is increased in premature infants requiring mechanical ventilation. (a) Lung H&E staining was performed in control infants who born at 24 weeks gestation and lived 3 hours (left), and short-term ventilated infant (born at 24 weeks gestation, lived 10 days) (right). Lungs in control infants are in the late canalicular/early saccular stage of development characterized by relatively large-sized acini, separated by relatively thin septa containing a well-developed capillary network. Compared with control lungs, the septa are wider and more cellular, the more irregular acini are lined by reactive epithelial cells, and filled with alveolar macrophages in short-term ventilated infants. Focal interstitial hemorrhages are also noted. Original magnification ×200. (b) Immunofluorescence was carried out to detect lamin b1 expression in lungs of premature infants requiring mechanical ventilation. (c) Cells lacking nuclear lamin b1 were counted and normalized to total numbers of nuclei. Arrows denote cells lacking nuclear lamin b1. Bar size: 25 µm. Data are expressed as mean ± SEM. N=5 subjects per group. Source data are provided as a Source Data file. T-test was used for comparison. $^{**}P<0.01$ *vs* control group.

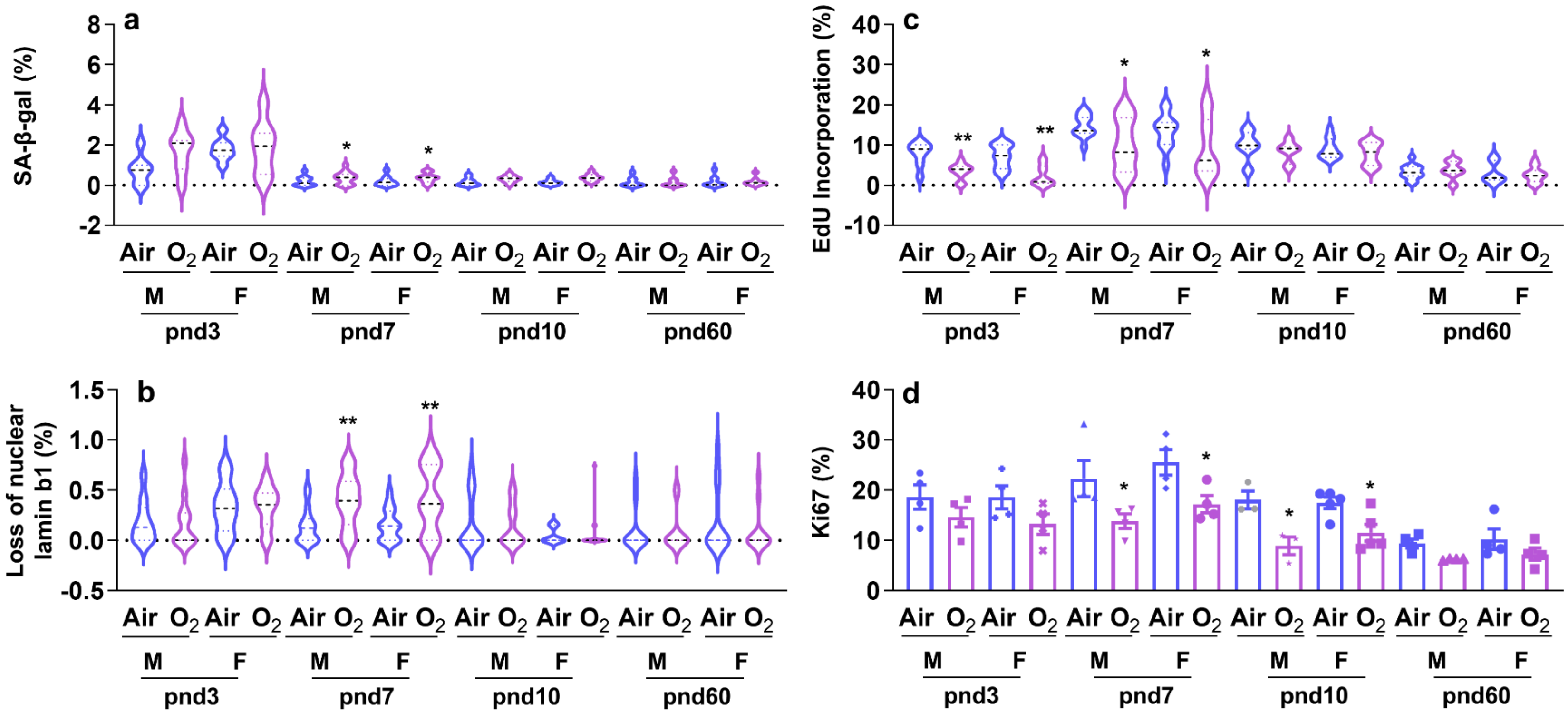

Supplementary Figure 2. No differences in lung senescence between male and female mice exposed to hyperoxia as neonates. C57BL/6J neonatal mice (<12 h old) were exposed to air or hyperoxia (>95% O_2) for 3 days followed by air recovery until pnd7, pnd10 and pnd60. Lung SA-β-gal activity (a), lamin b1 immunofluorescence (b), EdU incorporation assay (c) and Ki67 staining (d) were performed in both male and female mice. Data are expressed as mean ± SEM. N=4-5 mice per group. Source data are provided as a Source Data file. One-way ANOVA followed by Tukey post-test was used for multiple comparisons. $^{*}P<0.05$, $^{**}P<0.01$ *vs* corresponding air group.

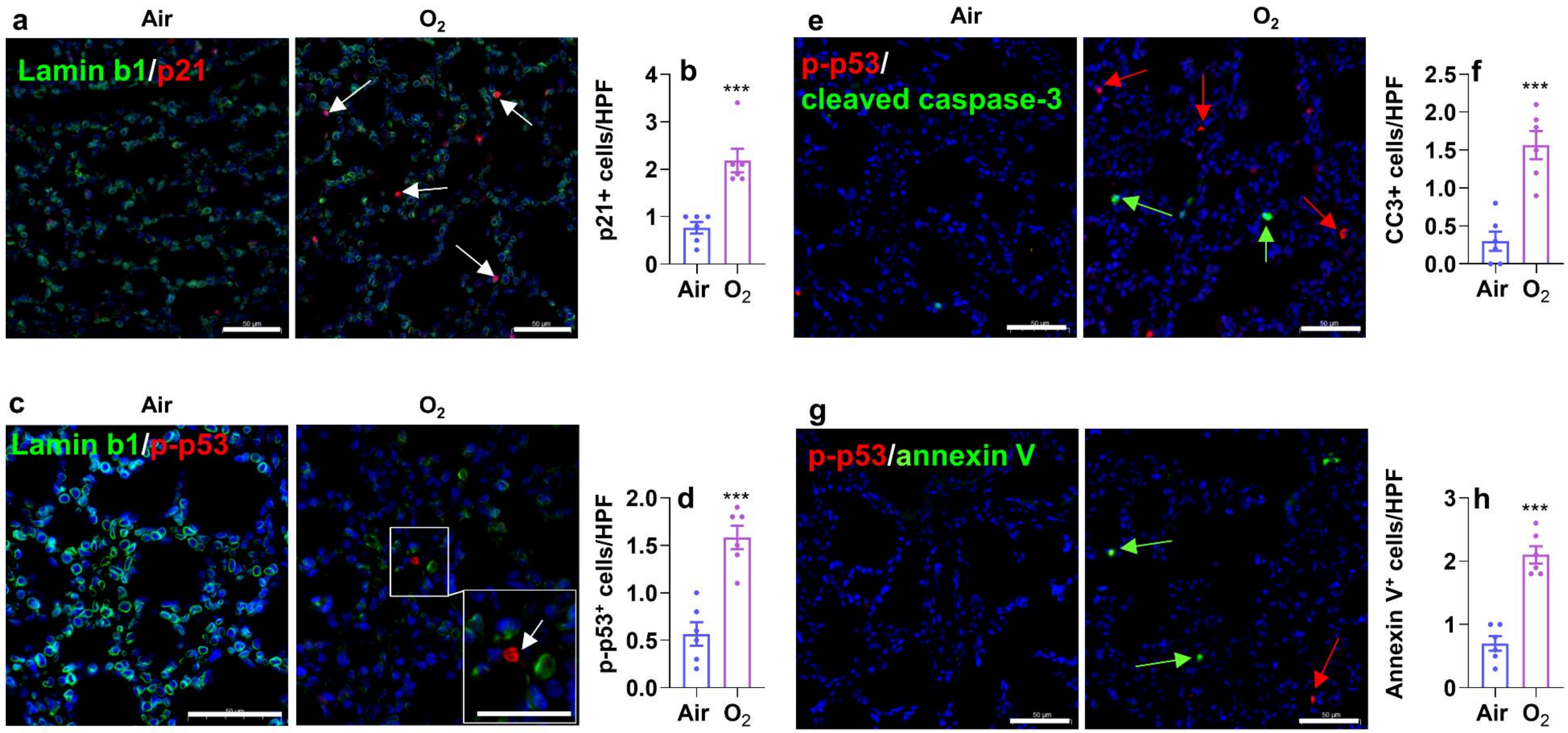

Supplementary Figure 3. p21 or phosphor-p53 positive cells lack lamin b1, and no colocalization of phosphor-p53 with apoptotic markers in the lung of mice exposed to hyperoxia as neonates. C57BL/6J neonatal mice (<12 h old) were exposed to air or hyperoxia (>95% O_2) for 3 days followed by air recovery until pnd7. (a-d) Immunofluorescence was performed to detect the colocalization of lamin b1 with p21 (a, b), and phosphor-p53 (c, d) in the lung. The number of $p21^+$ (b) or p-$p53^+$ cells (d) were counted in 3 randomly selected high-power fields (HPF) for each sample. (e-h) Immunofluorescence was carried out to detect the colocalization of phosphor-p53 with cleaved caspase-3 (CC3) or annexin V. The number of $CC3^+$ (f) or annexin V^+ cells (h) were counted in 3 randomly selected high-power fields (HPF) for each sample. Bar size: 50 µm. Data are expressed as mean ± SEM. N=4-6 mice per group. Source data are provided as a Source Data file. T-test was used for companion. $^{***}P<0.001$ *vs* air group.

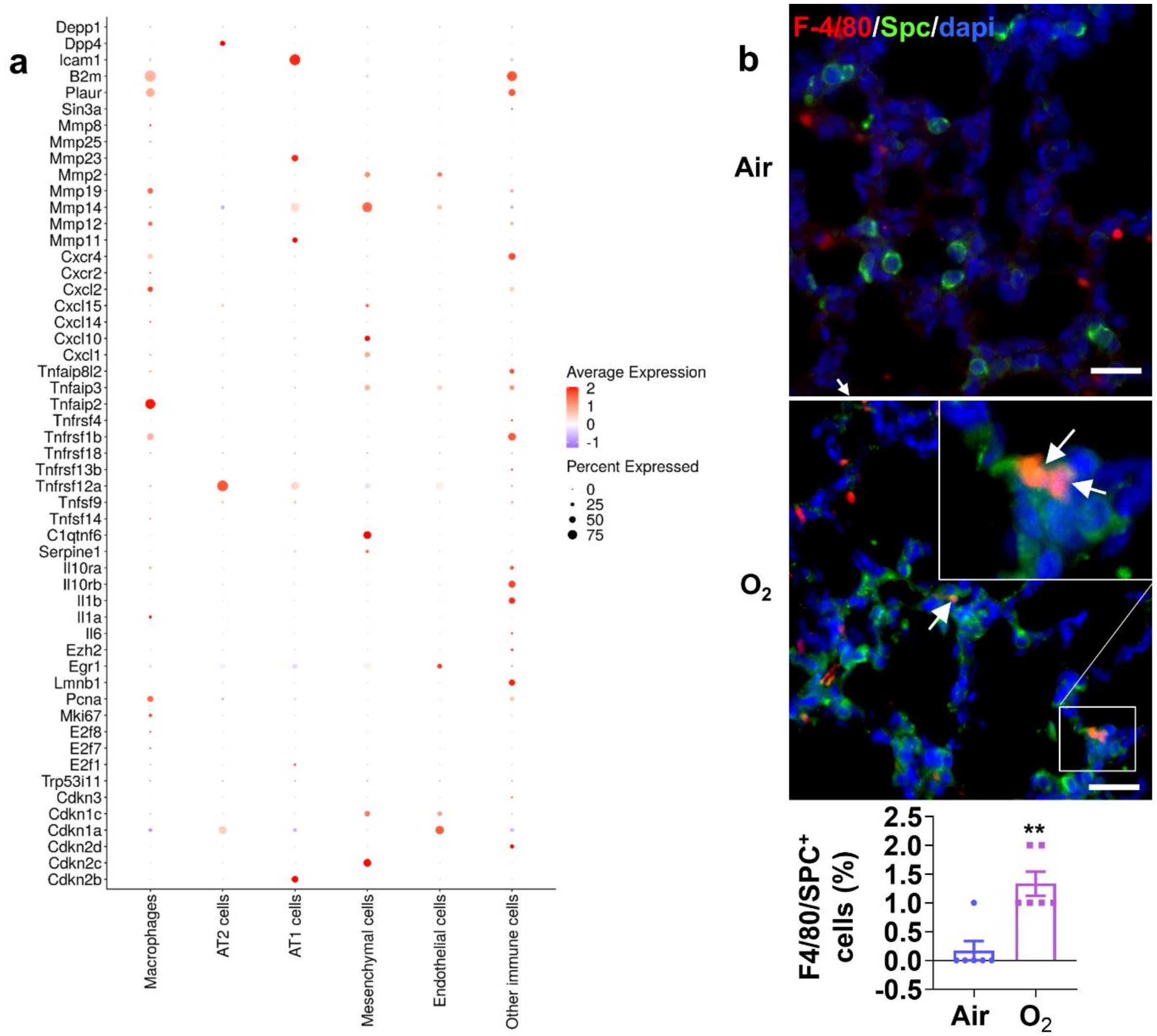

Supplementary Figure 4. Neonatal hyperoxia causes cell-specific expression of SASP genes in the lung. C57BL/6J neonatal mice (<12 h old) were exposed to air or hyperoxia (95% O_2) for 3 days. Some mice were then allowed to recover in room air until pnd7. scRNA-seq was performed in C_{12}FDG-sorted lung single cells from hyperoxia group at pnd7. (a) Gene expression among different types of cells in the C_{12}FDG-sorted lung single cells. Dot size is proportional to the percentage of cells expressing each gene. (b) Immunofluorescence was performed to detect the co-localization of F4-80 and pro-SPC in the lung of mice exposed to hyperoxia as neonates at pnd7. Bar size: 50 µm. Data are expressed as mean ± SEM. N=6 mice per group. Source data are provided as a Source Data file. T-test was used for companion in panel b. ***P*<0.01 *vs* air group.

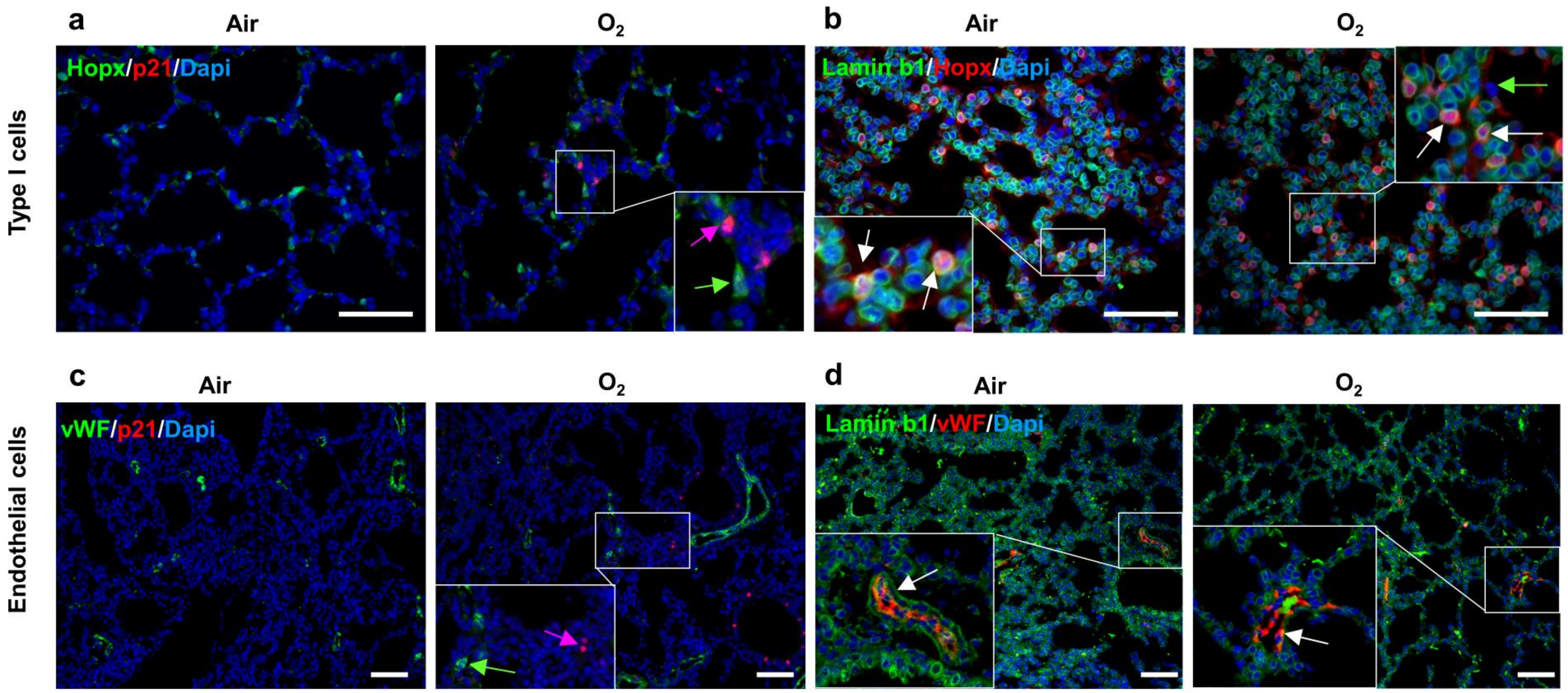

Supplementary Figure 5. No senescent type I or endothelial cells are observed in the lung of mice exposed to hyperoxia. C57BL/6J neonatal mice (<12 h old) were exposed to air or hyperoxia (>95% O_2) for 3 days followed by air recovery until pnd7. Immunofluorescence was performed to detect the co-localization of p21 (a, c) and lamin b1 (b, d) with Hopx (a, b), and vWF (c, d). Bar size: 50 µm. Representative images from three mice per group.

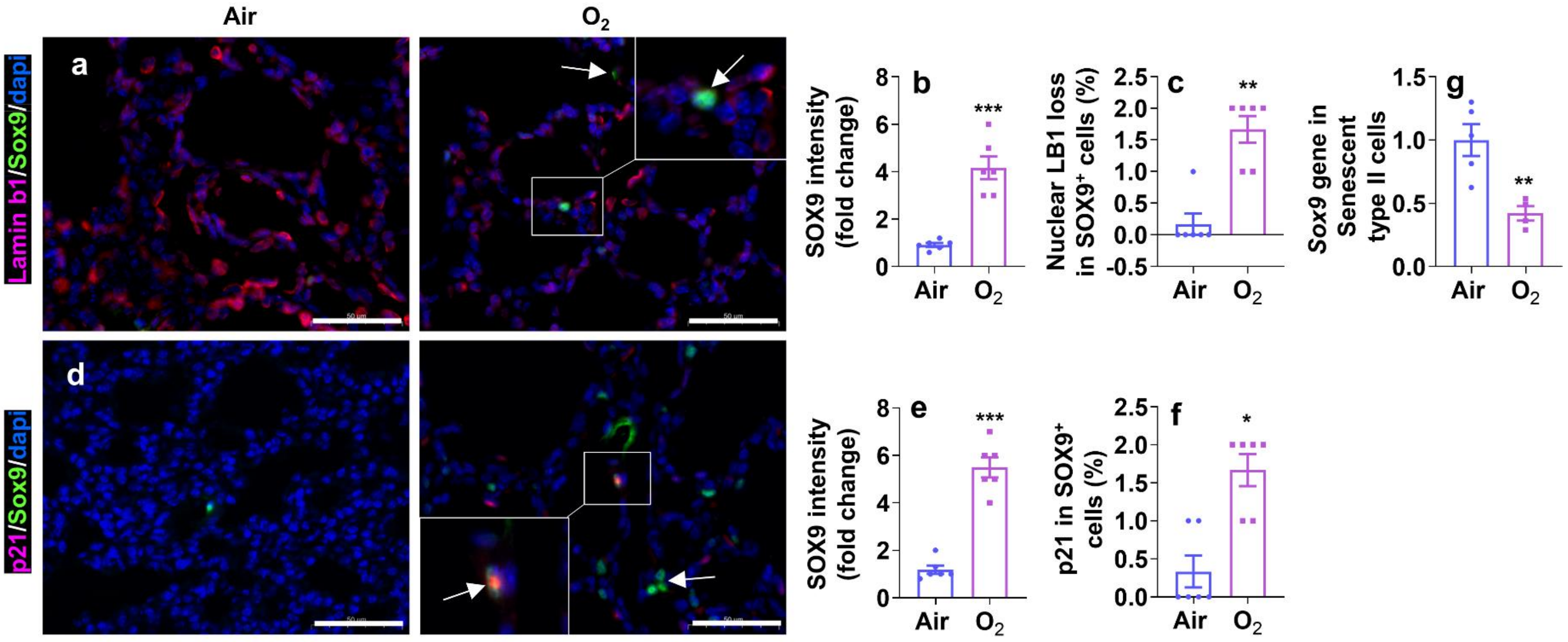

Supplementary Figure 6. Neonatal hyperoxia causes senescence in Sox9 positive cells but decreases Sox9 gene expression in senescent type II cells. C57BL/6J neonatal mice (<12 h old) were exposed to air or hyperoxia (95% O_2) for 3 days followed by air recovery until pnd7. (a) Immunofluorescence was performed to determine co-localization of loss of nuclear lamin b1 (LB1) with Sox9 in the lung. (b) Sox9 immunofluorescence was determined using Image J. (c) Cells lacking nuclear LB1 but positive for Sox9 were counted and normalized to total numbers of nuclei. (d) Immunofluorescence was performed to determine co-localization of p21 with Sox9 in the lung. (e) Sox9 immunofluorescence was determined using Image J. (f) Cells with co-localization of Sox9 and p21 were counted and normalized to total numbers of nuclei. (g) *Sox9* gene expression was reduced in isolated senescent type II cells from hyperoxia-exposed mice compared to non-senescent type II cells isolated from air control mice. Bar size: 50 µm. Data are expressed as mean ± SEM. N=4-6 mice per group. Source data are provided as a Source Data file. T-test was used for companion. $^{*}P<0.05$, $^{**}P<0.01$, $^{***}P<0.001$ *vs* air group.

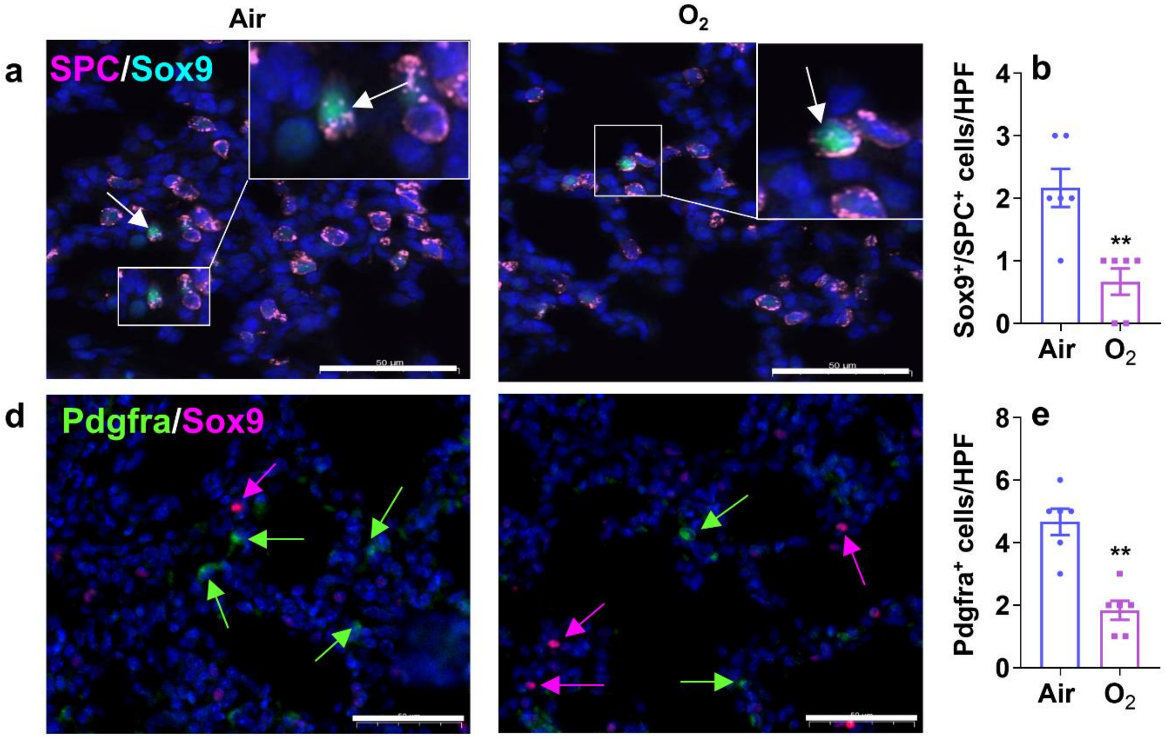

Supplementary Figure 7. Neonatal hyperoxia decreases Sox9 positive type II cells. C57BL/6J neonatal mice (<12 h old) were exposed to air or hyperoxia (95% O_2) for 3 days followed by air recovery until pnd7. (A) Immunofluorescence was performed to determine co-localization of Sox9 with SPC (a, b) and Pdgfra (c, d) in the lung. (b, d) The number of $Sox9^+/SPC^+$, or $Pdgfra^+$ cells were counted in 3 randomly selected high-power fields (HPF) for each sample in the lung. Bar size: 50 µm. Data are expressed as mean ± SEM. N=5-6 mice per group. Source data are provided as a Source Data file. T-test was used for companion. $^*P<0.05$, $^{**}P<0.01$ *vs* air group.

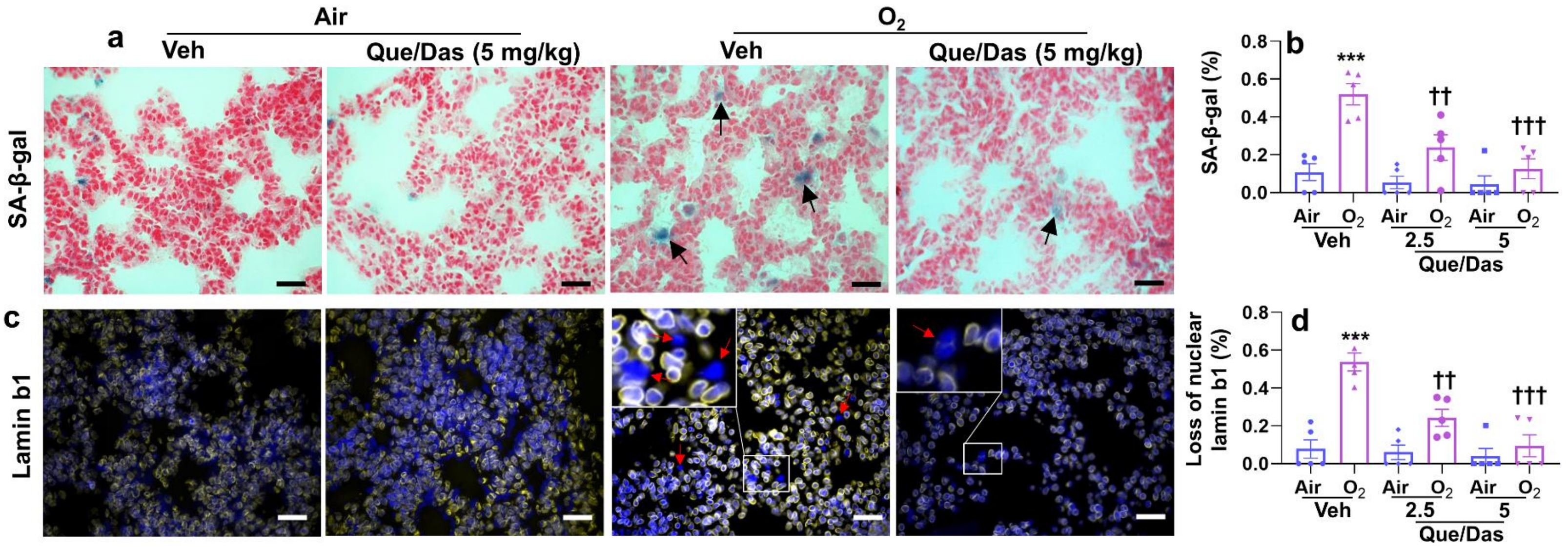

Supplementary Figure 8. Senolytic drug combination, dasatinib plus quercetin, attenuates neonatal hyperoxia-induced lung senescence. C57BL/6J neonatal mice (<12 h old) were exposed to air or hyperoxia (>95% O_2) for 3 days followed by air recovery until pnd7. Quercetin (Que, Q)/dasatinib (Das, D) (2.5 and 5 mg/kg) were intraperitoneally injected into mice at pnd4 and pnd6. SA-β-gal activity (a, b) and loss of nuclear lamin b1 (c, d) were assessed in the lung at pnd7 after Que/Das treatment. Bar size: 25 µm. Data are expressed as mean ± SEM. N=7 mice per group. Source data are provided as a Source Data file. One-way ANOVA followed by Tukey post-test was used for multiple comparisons. $^{***}P<0.001$ *vs* corresponding Air group. $^{††}P<0.01$, $^{†††}P<0.001$ *vs* corresponding Veh/O_2 group.

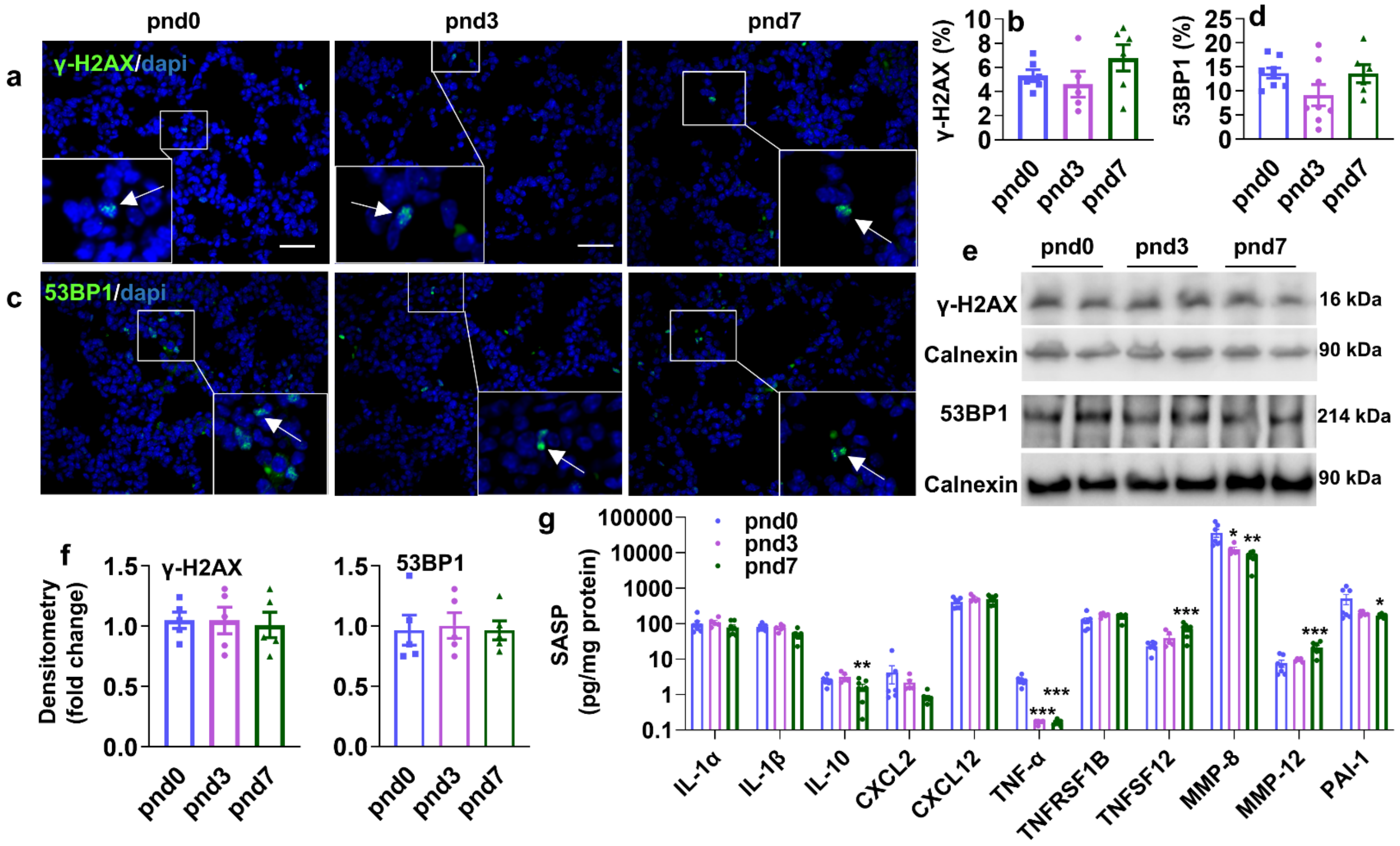

Supplementary Figure 9. No DNA damage, and SASP factors are detected during the saccular stage of lung development. (a-d) Immunofluorescence of γH2AX (a, b) and 53BP1 (c, d) was performed in the lung of mice at pnd0, pnd3 and pnd7 under normoxia. Cells positive for γH2AX (b) and 53BP1 (d) were counted and normalized to total numbers of nuclei in the lung. Arrows denote γH2AX or 53BP1 positive cells. Bar size: 25 µm. (e-f) Western blot was performed to determine the levels of γH2AX and 53BP1 in mouse lungs at pnd0, pnd3 and pnd7. (g) Luminex assay was performed to evaluate the SASP factors in the lung at pnd0, pnd3 and pnd7 under normoxia. Data are expressed as mean ± SEM. N=7 mice per group. Source data are provided as a Source Data file. One-way ANOVA followed by Tukey post-test was used for multiple comparisons. $^{*}P<0.05$, $^{**}P<0.01$, $^{***}P<0.001$ *vs* pnd0.

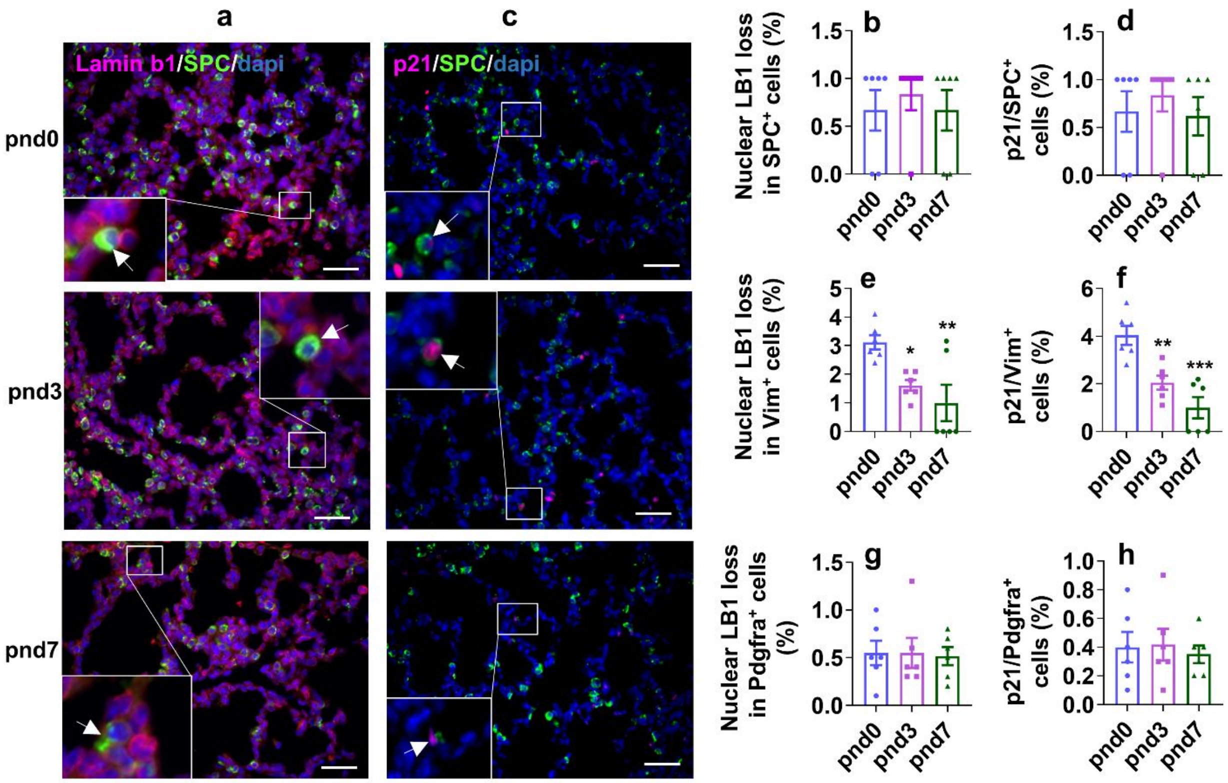

Supplementary Figure 10. No senescence markers in type II or Pdgfra$^+$ cells are detected during the saccular stage of lung development. (a-d) Immunofluorescence was performed to detect nuclear lamin b1 (LB1) loss and p21 expression in pro-SPC positive cells in the lung of mice at pnd0, pnd3 and pnd7 under normoxia. The number of nuclear lamin b1 loss in pro-SPC positive cells (b) and p21$^+$/pro-SPC$^+$ cells (d) were counted and normalized to total numbers of nuclei in the lung. (e-h) Immunofluorescence was performed to detect nuclear lamin b1 loss and p21 expression in vimentin (Vim, e-f) and Pdgfra (g-h) positive cells in the lung of mice at pnd0, pnd3 and pnd7 under normoxia. The number of nuclear lamin b1 loss in Vim (e) and Pdgfra (g) positive cells, p21$^+$/Vim$^+$ (f), p21$^+$/Pdgfra$^+$ (h) cells were counted and normalized to total numbers of nuclei in the lung. Arrows denote pro-SPC$^+$ cells lacking lamin b1 but expressing p21. Bar size: 50 µm. Data are expressed as mean ± SEM. N=5-6 mice per group. Source data are provided as a Source Data file. One-way ANOVA followed by Tukey post-test was used for multiple comparisons. $^*P<0.05$, $^{**}P<0.01$, $^{***}P<0.001$ *vs* pnd0.

Supplementary Table 1. **Clinical characteristics of subjects and patients**.

	Control (n = 5)	Short-term ventilated (n = 5)
Age at birth, week*	24.2 ± 1.6	24.4 ± 0.5
Postnatal age, day	<1	10.2 ± 4.0
Corrected age at death, week*	24.4 ± 1.8	25.6 ± 1.1
Sex	4M/1F	3M/2F
Body weight at autopsy, gram	746 ± 286	720 ± 198
Clinical/autopsy diagnosis	abruption (2); PROM/abruption (1); acute chorioamnionitis + sepsis (1); extreme prematurity, COD otherwise undetermined (1)	early BPD with complications of prematurity, including sepsis (2), sepsis/pneumonia (2), NEC/pneumonia (1)

Definition of abbreviations: BPD = bronchopulmonary dysplasia; COD = cause of death; F = female; M = male; NEC = necrotizing enterocolitis; PROM = premature rupture of membranes. Values represent means ± SD of (n) patients.

*Age and corrected age reflect postmenstrual age.

Supplementary Table 2. Antibodies and probes used in this study

Antibodies or probes	Name	Cat#	Dilution	Company
Antibody	53BP1	NB100-304	1:100	Novus
	8-oxo-DG	4354-MC-050	1:100	R&D System
	Pdgfra	AF-1062-SP	1:100	Novus Biologicals
	Calnexin	ADI-SPA-860-F	1:100	Enzo
	Pro-SPC	sc-518029	1:100	Santa Cruz
	Lamin b1	Ab16048/sc-377000	1:100	Abcam/Santa Cruz
	Cleaved caspase 3	9661	1:100	Cell Signaling
	F4/80	Ab6640	1:100	Abcam
	β-actin	Ab8227	1:100	
	p21	Ab107099	1:50	
	p53	Ab31333	1:1000 (WB), 1:100 (IF)	
	Vimentin	Ab92547	1:100	
	Sox9	Ab185230	1:100	
	vWF	Ab201336	1:100	
	Annexin V	Ab14196	1:100	
	Ki67	Ab16667	1:100	
	γH2AX	Ab26350	1:50	
	Alexa Fluor 488 goat anti-rabbit IgG	A11034	1:250	Thermo Fisher Scientific
	Alexa Fluor 488 goat anti-mouse IgG	A11001	1:250	
	Alexa Fluor 594 rabbit anti-goat IgG	A11080	1:250	
	Alexa Fluor 594 goat anti-rabbit IgG	A11072	1:250	
Probe	p16	Mm00494449_m1		ThermoFisher
	p21	Mm04205640_g1		
	Sox9	Mm00448840_m1		
	18S	Hs99999901_s1		
