# Supplementary material for: Timing and cell specificity of senescence drives postnatal lung development and injury
Source: Nat Commun. 2023 Jan 17;14:273. doi: 10.1038/s41467-023-35985-4 (PMC9845377; doi:10.1038/s41467-023-35985-4)
Supplement: Supplementary file 3 — Description of Additional Supplementary Files [file 41467_2023_35985_MOESM3_ESM.pdf]

Description of Additional Supplementary Files

Supplementary Figures and Supplementary Tables

Supplementary Data

Supplementary Data 1: SASP genes in C12FGD-sorted cells from hyperoxia-exposed mice vs cells in air (Air) group

Supplementary Data 2: Marker genes in C12FDG-sorted cells vs cells without C12FDG sorting from hyperoxia-exposed mice at pnd7

Supplementary Data 3: SASP genes in different clusters of C12FDG-sorted cells from hyperoxia-exposed mice at pnd7

Supplementary Data 4: M1 and M2 marker gene expression in different clusters of C12FDG postive macrophages at pnd7

Supplementary Data 5: Type II cell marker genes in C12FGD-sorted cells from hyperoxia-exposed mice at pnd7

Supplementary Data 6: Phagocytosis regulating genes in C12FGD-sorted cells from hyperoxia-exposed mice at pnd7

Supplementary Data 7: Gene in C12FGD-sorted cells from hyperoxia-exposed mice vs cells without C12FGD sorting from normoxic mice at pnd7

Supplementary Data 8: Genes in lung cells from hyperoxia vs air-exposed mice without C12FDG sorting at pnd7

Supplementary Data 9: Marker genes in different clusters of C12FGD-sorted cells from hyperoxia-exposed mice at pnd7

Source Data

Redacted Women and Infants Hospital post-mortem consent form
