# Supplementary material for: Timing and cell specificity of senescence drives postnatal lung development and injury
Source: Nat Commun. 2023 Jan 17;14:273. doi: 10.1038/s41467-023-35985-4 (PMC9845377; doi:10.1038/s41467-023-35985-4)
Supplement: Supplementary file 13 — Reporting Summary [file 41467_2023_35985_MOESM13_ESM.pdf]

nature portfolio

Corresponding author(s): Phyllis A. Dennery

Last updated by author(s): 2022-12-5

Reporting Summary

Nature Portfolio wishes to improve the reproducibility of the work that we publish. This form provides structure for consistency and transparency in reporting. For further information on Nature Portfolio policies, see our Editorial Policies and the Editorial Policy Checklist.

Statistics

For all statistical analyses, confirm that the following items are present in the figure legend, table legend, main text, or Methods section.

n/a	Confirmed	
☐	☒	The exact sample size (*n*) for each experimental group/condition, given as a discrete number and unit of measurement
☐	☒	A statement on whether measurements were taken from distinct samples or whether the same sample was measured repeatedly
☐	☒	The statistical test(s) used AND whether they are one- or two-sided<br>*Only common tests should be described solely by name; describe more complex techniques in the Methods section.*
☒	☐	A description of all covariates tested
☒	☐	A description of any assumptions or corrections, such as tests of normality and adjustment for multiple comparisons
☐	☒	A full description of the statistical parameters including central tendency (e.g. means) or other basic estimates (e.g. regression coefficient) AND variation (e.g. standard deviation) or associated estimates of uncertainty (e.g. confidence intervals)
☐	☒	For null hypothesis testing, the test statistic (e.g. *F*, *t*, *r*) with confidence intervals, effect sizes, degrees of freedom and *P* value noted<br>*Give P values as exact values whenever suitable.*
☒	☐	For Bayesian analysis, information on the choice of priors and Markov chain Monte Carlo settings
☒	☐	For hierarchical and complex designs, identification of the appropriate level for tests and full reporting of outcomes
☒	☐	Estimates of effect sizes (e.g. Cohen's *d*, Pearson's *r*), indicating how they were calculated

Our web collection on statistics for biologists contains articles on many of the points above.

Software and code

Policy information about availability of computer code

Data collection	The Zeiss Axiovert 200M Fluorescence Microscope system was controlled by AxioVision 4.8.2 software. Immuno-fluorescence micrographs were performed on a Zeiss Axiovert 200M Fluorescence Microscope and processed using ImageJ (v 1.53t). Quality control of scRNA-seq data was performed using the R package Seurat v 3.2.1. All of the Seurat code and the Docker file for the environment is available on Zenodo at https://zenodo.org/record/7401907.
Data analysis	Wilcoxon rank sum test was used to identify marker genes of scRNA-seq data. SCTransform (v 0.3.5) was used for normalization of all scRNA-seq datasets. Statistical analyses were performed using GraphPad Prism 9.4.0. The results were expressed as mean ± SEM. The t-test was used for detecting statistical significance of the differences between means of two groups after checking the normality of data. The statistical significance of the differences among groups was evaluated by using one-way ANOVA for overall significance, followed by Tukey’s multiple comparisons test.

For manuscripts utilizing custom algorithms or software that are central to the research but not yet described in published literature, software must be made available to editors and reviewers. We strongly encourage code deposition in a community repository (e.g. GitHub). See the Nature Portfolio guidelines for submitting code & software for further information.

Data

Policy information about availability of data

All manuscripts must include a data availability statement. This statement should provide the following information, where applicable:

- Accession codes, unique identifiers, or web links for publicly available datasets
- A description of any restrictions on data availability
- For clinical datasets or third party data, please ensure that the statement adheres to our policy

The scRNA-seq generated in this study have been deposited in the Gene Expression Omnibus database under primary accession code GSE207866. All other data needed to evaluate the conclusions of the study are present in the paper or in the Supplementary files. Source data are provided with this paper.

Human research participants

Policy information about studies involving human research participants and Sex and Gender in Research.

Reporting on sex and gender	We included sex and gender information in the manuscript. However, we did not perform sex-based analysis due to limited number of samples used in the study.
Population characteristics	We provided clinical characteristics of two groups of subjects and patients.
Recruitment	Not applicable.
Ethics oversight	Women and Infants Hospital, Providence RI

Note that full information on the approval of the study protocol must also be provided in the manuscript.

Field-specific reporting

Please select the one below that is the best fit for your research. If you are not sure, read the appropriate sections before making your selection.

☒ Life sciences ☐ Behavioural & social sciences ☐ Ecological, evolutionary & environmental sciences

For a reference copy of the document with all sections, see nature.com/documents/nr-reporting-summary-flat.pdf

Life sciences study design

All studies must disclose on these points even when the disclosure is negative.

Sample size	N=8-10 mice per group were chosen based on our previous publication (PMID: 32815166), which generated 80% power to detect the difference. This allows n=4 or 5 male and females mice each group used for evaluating sex differences of hyperoxia-induced senescence.
Data exclusions	No data were excluded from the analysis.
Replication	All attempts at replication were successful for the experiments. The experiments were performed independently. All light microscopy experiments were done in triplicate. For immunofluorescence, at least three individual slices were analyzed.
Randomization	Animals were randomized to allocated into air, hyperoxic exposure and treatments. Human samples were randomly picked up from our tissue bank for staining.
Blinding	A subset of the data sets (e.g., immunostaining and lung morphometry) has been analyzed in a single-blind approach. The investigators were blinded to group allocation during data collection, but not blinded for statistical analysis. The scRNA-seq data were analyzed without a blinding approach. This is because we need to know senescence group (O2/pnd7) to evaluate cluster-specific gene expression and compare this with air/pnd7 group.

Reporting for specific materials, systems and methods

We require information from authors about some types of materials, experimental systems and methods used in many studies. Here, indicate whether each material, system or method listed is relevant to your study. If you are not sure if a list item applies to your research, read the appropriate section before selecting a response.

Materials & experimental systems

n/a	Involved in the study	
☐	☒	Antibodies
☒	☐	Eukaryotic cell lines
☒	☐	Palaeontology and archaeology
☐	☒	Animals and other organisms
☒	☐	Clinical data
☒	☐	Dual use research of concern

Methods

n/a	Involved in the study	
☒	☐	ChIP-seq
☒	☐	Flow cytometry
☒	☐	MRI-based neuroimaging

Antibodies

Antibodies used

Detailed information about the antibodies used in this study are provided in Supplementary Table 2.

Validation

Antibody validation:
53BP1 antibody: Application statement in manufacturer's website as following: https://www.novusbio.com/products/53bp1-antibody_nb100-304
8-oxo-DG antibody: Application statement in manufacturer's website as following: https://www.rndsystems.com/products/8-oxo-dg-antibody-15a3_4354-mc-050
Pdgfra antibody: Application statement in manufacturer's website as following: https://www.novusbio.com/products/pdgfr-alpha-antibody_af1062
Calnexin antibody: Application statement in manufacturer's website as following: https://www.enzolifesciences.com/ADI-SPA-860/calnexin-polyclonal-antibody/
Pro-SPC antibody: Application statement in manufacturer's website as following: https://datasheets.scbt.com/sc-518029.pdf
Lamin b1 antibody: Application statement in manufacturer's website as following: https://www.abcam.com/lamin-b1-antibody-nuclear-envelope-marker-ab16048.html
Lamin b1 antibody: Application statement in manufacturer's website as following: https://datasheets.scbt.com/sc-377000.pdf
Cleaved caspase-3 antibody: Application statement in manufacturer's website as following: https://www.cellsignal.com/products/primary-antibodies/cleaved-caspase-3-asp175-antibody/9661
F4/80 antibody: Application statement in manufacturer's website as following: https://www.abcam.com/f480-antibody-cia3-1-macrophage-marker-ab6640.html
β-actin antibody: Application statement in manufacturer's website as following: https://www.abcam.com/beta-actin-antibody-ab8227.html
p21 antibody: Application statement in manufacturer's website as following: https://www.abcam.com/p21-antibody-hugo291-ab107099.html
p53 antibody: Application statement in manufacturer's website as following: https://www.abcam.com/p53-antibody-ab31333.html
Vimentin antibody: Application statement in manufacturer's website as following: https://www.abcam.com/vimentin-antibody-epr3776-cytoskeleton-marker-ab92547.html
Sox9 antibody: Application statement in manufacturer's website as following: https://www.abcam.com/sox9-antibody-epr14335-ab185230.html
vWF antibody: Application statement in manufacturer's website as following: https://www.abcam.com/von-willebrand-factor-antibody-3e2d10--vwf635-ab201336.html
Annexin V antibody: Application statement in manufacturer's website as following: https://www.abcam.com/annexin-vanxa5-antibody-ab14196.html
Ki67 antibody: Application statement in manufacturer's website as following: https://www.abcam.com/ki67-antibody-sp6-ab16667.html
γH2AX antibody: Application statement in manufacturer's website as following: https://www.abcam.com/gamma-h2ax-phospho-s139-antibody-9f3-ab26350.html
Alexa Fluor 488 goat anti-rabbit IgG antibody: Application statement in manufacturer's website as following: https://www.thermofisher.com/order/genome-database/dataSheetPdf?producttype=antibody&productsubtype=antibody_secondary&productId=A-11034&version=271
Alexa Fluor 488 goat anti-mouse IgG antibody: Application statement in manufacturer's website as following: https://www.thermofisher.com/order/genome-database/dataSheetPdf?producttype=antibody&productsubtype=antibody_secondary&productId=A-11001&version=271
Alexa Fluor 594 rabbit anti-goat IgG antibody: Application statement in manufacturer's website as following: https://www.thermofisher.com/order/genome-database/dataSheetPdf?producttype=antibody&productsubtype=antibody_secondary&productId=A-11080&version=271
Alexa Fluor 594 goat anti-rabbit IgG antibody: Application statement in manufacturer's website as following: https://www.thermofisher.com/order/genome-database/dataSheetPdf?producttype=antibody&productsubtype=antibody_secondary&productId=A-11072&version=271

Animals and other research organisms

Policy information about studies involving animals; ARRIVE guidelines recommended for reporting animal research, and Sex and Gender in Research

Laboratory animals	C57BL/6J mice with postnatal day (pnd) 0, pnd3, pnd7, pnd10 and pnd60 were used in the study.
Wild animals	No wild animals were used in the study.
Reporting on sex	Both male and female mice were used in the study. Sex-based analysis on lung senescence was performed.
Field-collected samples	No field-collected samples were used.
Ethics oversight	All animal experiments were reviewed and approved by the Institutional Animal Care and Use Committee of Brown University (IACUC: 21-08-0003).

Note that full information on the approval of the study protocol must also be provided in the manuscript.
